# Synthesis and Biological Activity of Novel Phenyltriazolinone Derivatives

**DOI:** 10.3390/molecules15129024

**Published:** 2010-12-09

**Authors:** Qiongyou Wu, Guodong Wang, Shaowei Huang, Long Lin, Guangfu Yang

**Affiliations:** Key Laboratory of Pesticide and Chemical Biology of Ministry of Education, College of Chemistry, Central China Normal University, Wuhan 430079, Hubei, China

**Keywords:** phenyltriazolinones, strobilurin, herbicidal activity, protoporphyrinogen oxidase

## Abstract

Phenyltriazolinones are one of the most important classes of herbicides targeting the protoporphyrinogen oxidase enzyme. A series of triazolinone derivatives containing a strobilurin pharmacophore were designed and synthesized with the aim of discovering new phenyltriazolinone analogues with high activity. The herbicidal activity of the synthesized compounds was assayed and some of the test compounds displayed moderate herbicidal activity at 150 g ai/ha.

## 1. Introduction

Triazolinones are attractive building blocks of considerable interest because of their unique properties and wide range of biological properties [1,2,3,4,5,6], such as human neurokinin-1 receptor antagonist [7] and angiotensin AII receptor antagonist [8,9] activity. In the past two decades, research on triazolinone herbicides has attracted continuous interest [10,11,12,13,14] since the discovery of sulfentrazone, the first commercialized phenyltriazolinone herbicide with excellent pre-emergence control of several broadleaf weeds as well as several selected grass weeds for the soybean market [15]. Carfentrazone-ethyl, a second commercial herbicide introduced by FMC only a few years after sulfentrazone was marketed, showed excellent post-emergence cereal and corn herbicidal activities [16]. Nowadays, phenyltriazolinone herbicides play an important role in the herbicide market. The mode of action of sulfentrazone and carfentrazone-ethyl is the inhibition of protoporphyrinogen oxidase (Protox), which causes the accumulation of protoporphyrin IX (Proto IX), which is involved in the light-dependent formation of singlet oxygen responsible for membrane peroxidation [17,18]. This unique mode of action, that makes phenyltriazolinones safe, high efficient and environmentally benign herbicides, has been actively pursued and Protox inhibitors have been used very effectively for many decades, although a biotype of *Amaranthus tuberculatus* has recently evolved resistance to these herbicides via a codon deletion mutation that affects the binding of the inhibitors to the enzyme [19,20].

Structure-activity relationship analysis indicated that the 2,4,5-trisubstituted phenyl structure plays an important role for their herbicidal activities of phenyltriazolinones. Among the phenyl substitution patterns investigated, F or Cl at C-2 and Cl at C-4 was identified as crucial for most the active compounds, while a wide range of substitutions at C-5 were acceptable. Additionally, among the N-4 substituents investigated, the CHF_2_ group always gave the highest herbicidal activity, although the success of azafenidin indicated that groups other than CHF_2_ are also acceptable at N-4 [21]. 

**Figure 1:**
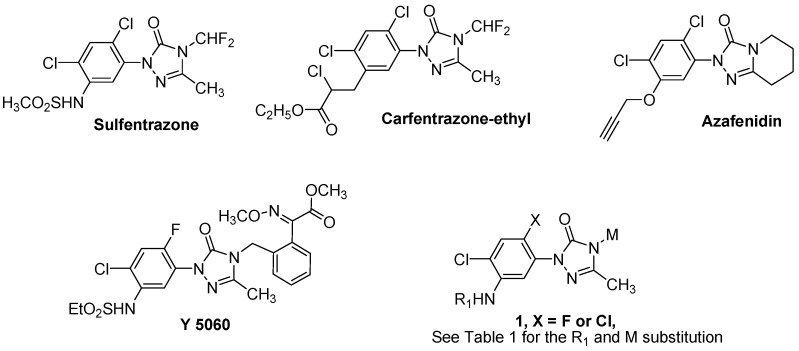
Structures of sulfentrazone, carfentrazone-ethyl, azafenidin, **Y5060** and the new compounds synthesized in this study.

The strobilurins are a class of fungicidal compounds which have been applied as agricultural disinfectants in many countries. Most active strobilurin compounds contain the same biologically active moiety, that is, an acrylate, an acetate, or an acetamide chemical group with the *E* configuration about the double bond in the toxophore moiety [22,23,24]. We envisaged that, if the strobilurin pharmacophore was introduced into the phenyltriazolinone scaffold, the resulting product (Figure 1, compounds **1**) should be an interesting lead structure for agrochemical development. In our previous studies [25], we have introduced the (*E*)-methyl 2-methoxyimino-2-*o*-tolylacetate toxophore into the N-4 position of sulfentrazone and the resulting compound **Y5060** showed comparable herbicidal activity at 75-150 g of active ingredient/ha with the commercial product sulfentrazone. On the basis of test results of herbicidal spectrum and crop selectivity, compound **Y5060** was verified as a promising candidate for further development as a postemergence herbicide. To further improve the herbicidal activity of this class of compounds and screen for valuable lead compounds, according to the bioisosteric principle, a series of new compounds were designed and synthesized.

## 2. Results and Discussion

The preparation of the key intermediates **4** starts with an appropriately substituted aniline which is converted into the corresponding phenylhydrazine by diazotization with NaNO_2_ in concentrated HCl solution at low temperature and subsequently reduced with SnCl_2_ [10]. The phenylhydrazines were then treated with a ketocarboxylic acid without separation to afford phenylhydrazone **3**, which undergoes a Schmidt rearrangement upon reaction with diphenylphosphoryl azide and is thus converted into the corresponding triazolinone **4**. Thereafter, the benzene ring of the intermediate **4** is nitrated with the common mixed H_2_SO_4_ and HNO_3 _nitration reagent and the nitro group was then reduced with iron powder to form the aromatic amine **6**, which is then treated with the corresponding sulfonyl chloride in the presence of a weak base such as triethylamine to provide sulfonyl amide **7**. It was noticed that the hydrogen on 4-N position of the triazolinone ring is also replaced during the treatment with the chloride.

**Scheme 1:**
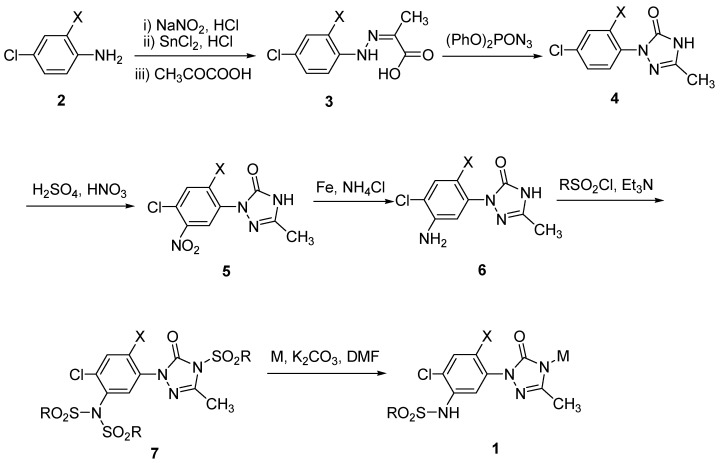
Synthetic route of the target compound **1**.

**Scheme 2:**
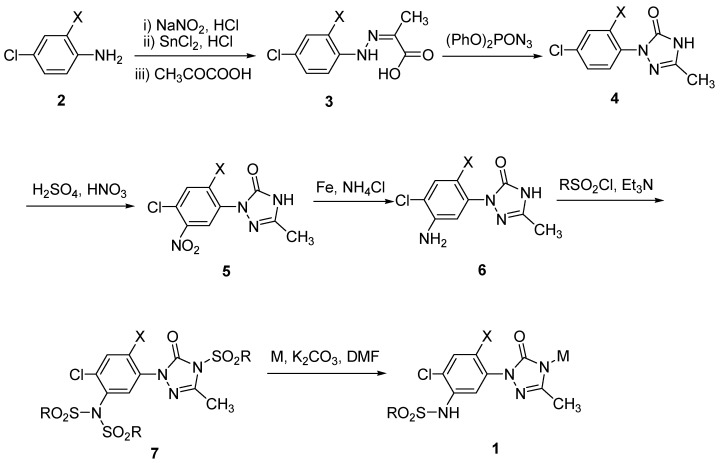
Synthesis of the intermediate M_1_.

Initially, we selected three typical strobilurin pharmacophores (M_1-3_) [26,27] as substituents to be introduced at the 4-N position of triazolinone **7** to investigate the effect of these substitution patterns on their herbicidal activity. The preparation of intermediate M_1_ was achieved in five steps with simple 2-*o*-tolylacetic acid as starting material (Scheme 2). 

The methyl 2-*o*-tolylacetate was treated with methyl formate in the presence of sodium methoxide to afford the mixed enol (*E/Z*), which can be methylated with dimethyl sulfate to give the desired *E*-isomer stereoselectively and successively brominated with NBS to afford the methoxymethyl acrylate M_1_ in excellent yield.

**Scheme 3:**
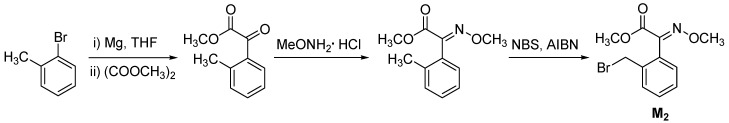
Synthesis of the intermediate M_2_.

For the synthesis of intermediate M_2 _(Scheme 3), the selected starting material was 2-bromotoluene, which was first converted into the Grignard reagent and then underwent nuclophilic addition with dimethyl oxalate to form methyl 2-oxo-2-*o*-tolylacetate, which after treatment with methoxylamine provided a mixed oxime. The *E/Z* ratio in the crude mixture was determined by ^1^H-NMR spectroscopy as ca. 1:1. After further column chromatography purification, the pure *E*-isomer was obtained in 51% yield. Similarly, the bromination of the *E*-oxime with 1.2 equiv. NBS afford target intermediate M_2_ smoothly.

**Scheme 4:**
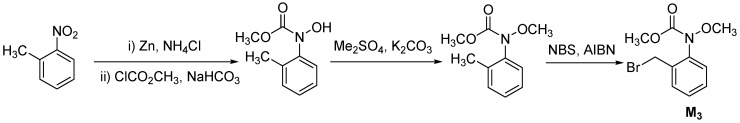
Synthesis of the intermediate M_3_.

The third intermediate M_3_ was prepared according to the procedure shown in Scheme 4. Firstly, 2-nitrotoluene was converted into *N*-hydroxy-2-methylbenzenamine by reduction with zinc in aqueous NH_4_Cl solution to give *N*-hydroxybenzenamine which undergoes nucleophilic substitution with methyl chloroformate to afford the *N*-acetylated product and subsequently the free *N*-hydroxy group was methylated with dimethyl sulfate to give the methoxycarbamate product, which was then transformed into intermediate M_3_ with NBS.

With these key intermediates in hand, we then investigated the coupling reaction of these intermediates M_1-3_ with the sulfonyl group protected phenyltriazolinone **7**. First, we tried a two-step synthetic procedure by treating compound **7** bearing three RSO_2_- groups with a base such as NaOH to obtain the 4-N deprotected product, which subsequently underwent nucleophilic substitution with intermediates M_1-3_ to give the desired products, albeit in very low yield (17 %). Meanwhile, if we combined the two-step reaction into a one-pot version, we found that the RSO_2_-group can be removed readily by treatment with a weak base such as K_2_CO_3_, after which the appropriate M substitution can be concomitantly introduced at the 4-N position of the triazolinone ring. Thus, compounds **1a**～**1q** were smoothly prepared in moderate to good yield (Table 1). 

**Table 1:** Structure of the synthesized target molecules **1**.

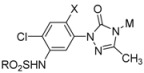					
No.	X	R	M^a^	m.p. (°C)	Yield (%)
**1a**	F	CH_3_-	M_1_	153-155	52
**1b**	F	CH_3_CH_2_-	M_1_	167-169	59
**1c**	F	Ph-	M_1_	187-190	39
**1d**	Cl	CH_3_-	M_1_	138-140	78
**1e**	Cl	CH_3_CH_2_-	M_1_	168-170	80
**1f**	Cl	Ph-	M_1_	194-196	48
**1g**	F	CH_3_-	M_2_	138-140	75
**1h**	F	Ph-	M_2_	164-166	46
**1i**	Cl	CH_3_-	M_2_	150-152	73
**1j**	Cl	CH_3_CH_2_-	M_2_	162-164	79
**1k**	Cl	Ph-	M_2_	190-192	44
**1l**	F	CH_3_-	M_3_	186-188	57
**1m**	F	CH_3_CH_2_-	M_3_	166-168	55
**1n**	F	Ph-	M_3_	oil	41
**1o**	Cl	CH_3_SO_2_-	M_3_	189-191	62
**1p**	Cl	CH_3_CH_2_SO_2_-	M_3_	174-176	70
**1q**	Cl	PhSO_2_-	M_3_	oil	46
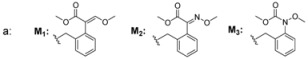					

The herbicidal activities of compounds **1a**～**1q** against monocotyledon weeds such as *Echinochloa crusgalli* (EC), *Digitaria sanguinalis* (DS), *Poa annua* (PA) and dicotyledon weeds such as *Brassica juncea (BJ), Amaranthus retroflexus (AR),* and *Eclipta prostrate (EP)* were evaluated according to a previously reported procedure [28]. Sulfentrazone was selected as a control. Most of the test compounds did not show the desired herbicidal activity at 150 g of ai/ha. However, some compounds present a certain degree of herbicidal activity. For example, compounds **1k**, **1l** and **1m** exhibited 70% activity against *E. prostrate,* compounds **1p** and **1q** displayed moderate herbicidal activity against *E. crusgalli*, *D. sanguinalis* and *P. annua*. In comparison, their herbicidal activity is lower than that of the lead compound **Y5060**. 

In conclusion, several strobilurin type pharmacophores were introduced to the 4-N position of the phenyl triazolinone scaffold with the view of preparing and screening potentially highly herbicidal lead compounds. The herbicidal activity of these synthesized compounds against six weeds was investigated. Unfortunately, they did not display desired weeds control potential compared to the lead compound **Y5060**. 

## 3. Experimental

### 3.1. General

Unless otherwise noted, all materials were commercially available and were used directly without further purification. All solvents were redistilled before use. ^1^H-NMR spectra were recorded at 400 MHz on a Mercury-Plus 400 spectrometer in CDCl_3_ (unless stated otherwise) with tetramethylsilane as the internal reference. MS spectra were determined using a Trace MS 2000 organic mass spectrometry. Elementary analyses were performed on a Vario EL III elementary analysis instrument. Melting points were taken on a Buchi B-545 melting point apparatus and uncorrected. 

### 3.2. General Procedure for the Preparation of Phenylhydrazines ***3***

To a cold solution of the appropriate 2,4-disubstituted benzenamine (0.10 mol) in concentrated HCl (100 mL), aqueous NaNO_2_ (8.5 g, 0.10 mol) was added dropwise during a period of 0.5 h under Ar. The result solution was stirred for a further 1 h at −9 °C, then a solution of SnCl_2_ in conc. HCl (40 mL) was added slowly over 40 min. After stirring for an another 0.5 h at −9 °C, the ice-salt bath was removed and the reactants were allowed to slowly warm to room temperature and then stirred at room temperature for 2 h. Then water (80 mL) and pyruvate (8.8 g, 0.05 mol) in water (80 mL) were added. The result reaction mixture was stirred for 30 min. The precipitate was collected and dried to give the phenylhydrazine **3**. **3a**, X = F, yield, 83%, m.p., 176-178 °C (lit: 172-173 °C [29]), **3b**, X = Cl, yield, 89%，m.p., 188-190 °C (lit: 193-194 °C [29]).

### 3.3. General Procedure for the Synthesis of Phenyltriazolinones ***4***

To a mixture of phenylhydrazone **3** (0.05 mol) and triethylamine (5.1 g, 0.05 mol) in toluene (30 mL) was slowly added diphenyl phosphorous azide (13.75 g, 0.05 mol). The mixture was refluxed until the reaction was complete according to the TLC monitoring. The reactants were cooled to room temperature and extracted with 1 M NaOH solution (50 mL). The water layer was separated and neutralized with concentrated HCl. The white precipitate was collected by filtration, washed with water and dried to afford the product **4**. **4a**, X = F, yield, 75%，m.p. 204-206 °C (lit: 201-203 °C [29]), ^1^H- NMR: δ 2.29 (s, 3H, CH_3_), 7.24 (q, 1H, ArH), 7.27 (d, 1H, ArH), 7.47 (t, 1H, ArH), 11.64 (s, 1H, NH); **4b**, X = Cl, yield, 92%，m.p. 179-181 °C (lit: 174-175 °C [14])，^1^H-NMR: δ 2.18 (s, 3H, CH_3_), 7.35 (q, 1H, ArH), 7.47 (d, 1H, ArH), 7.27 (d, 1H, ArH), 11.49 (s, 1H, NH).

### 3.4. General Procedure for the Synthesis of Nitrophenyltriazolinones ***5***

To a stirred solution of phenyltriazolinone **4** (5.00 mol) in concentrated H_2_SO_4_ (10 mL) was added concentrated HNO_3_ (0.45 g) slowly at 0 °C. After stirring for 0.5 h at this temperature, the mixture was allowed to warm to room temperature and stirred for a further 1 h, then the reaction mixture was poured into ice water and the precipitate was collected and dried to afford the desired product **5**. **5a**, X = F, yield, 95%,^ 1^H-NMR: δ 2.32 (s, 3H, CH_3_), 7.47 (d, 1H, *J* = 9.2 Hz, ArH), 8.30 (d, 1H, *J* = 6.8 Hz, ArH), 11.40 (s, 1H, NH); **5b**, X = Cl，yield, 98%，^1^H-NMR: δ 2.13 (s, 3H, CH_3_), 6.84 (s, 1H, ArH), 7.45 (s, 1H, ArH), 11.61 (s, 1H, NH).

### 3.5. General Procedure for the Synthesis of Aminophenyltriazolinones ***6***

A mixture of nitrophenyltriazolinone **5** (0.01 mol) and NH_4_Cl (0.55 g) in ethanol (25 mL) and water (3 mL) was refluxed for 0.5 h. Iron powder (1.68 g, 0.03 mol) was then added to the refluxing solution in several portions. The reaction was monitored by TLC until the starting material was consumed. The reaction mixture was filtrated through diatomite and washed with ethanol. The combined filtrate was concentrated to a half volume and the precipitate was collected to afford the product. **6a**, X = F, yield, 75%. ^1^H-NMR (DMSO): δ 2.15 (s, 3H, CH_3_), 5.41(s, 2H, NH_2_), 6.89 (d, 1H, *J* = 7.2 Hz, ArH), 7.32 (d, 1H, *J* = 10.4 Hz, ArH), 11.68 (s, 1H, NH); **6b**, X = Cl, yield, 84%, ^1^H-NMR (DMSO): δ 2.12 (s, 3H, CH_3_), 5.77 (s, 2H, NH_2_), 6.86 (s, 1H, ArH), 7.44 (s, 1H, ArH), 11.73 (s, 1H, NH).

### 3.6. General Procedure for the Synthesis of Compound ***7***

To a solution of compound **6** (0.01 mol) and triethylamine (0.03 mol) in CH_2_Cl_2_ (25 mL) was added dropwise the appropriate sulfonyl chloride (0.03 mol) at 0 °C. The reaction mixture was kept at 0 °C for a further 1.5 h and then washed with water. The combined organic phase was dried with NaSO_4_ and evaporated on a rotary evaporator. The residue was chromatographed on silica gel with ethyl acetate/petroleum ether (1:4) to give product **7** in a yield of 75%~85%.

### 3.7. General Procedure for the Preparation of Target Molecules ***1***

A mixture of the appropriate intermediate M (0.006 mol), compound **7** (0.005 mol) and anhydrous K_2_CO_3_ (0.015 mol) in DMF (20 mL) was stirred at room temperature until the reaction was complete according to TLC. The reaction mixture was poured into ice water (200 mL) and extracted with ethyl acetate. The combined organic phase was dried with NaSO_4_ and evaporated on a rotary evaporator. The residue was chromatographed on silica gel with ethyl acetate/petroleum ether (1:5) as eluent to give the target product. 

*(E)-methyl 2-(2-((1-(4-chloro-2-fluoro-5-(methylsulfonamido)phenyl)-3-methyl-5-oxo-1H-1,2,4-triazol-4(5H)-yl)methyl)phenyl)-3-methoxyacrylate* (**1a**): ^1^H-NMR: δ 1.92 (s, 3H, CH_3_), 3.11 (s, 3H, SO_2_CH_3_), 3.64 (s, 3H, OCH_3_), 3.88 (s, 3H, OCH_3_), 4.69(s, 1H, CH_2_), 4.98 (s, 1H, CH_2_), 6.80 (s, 1H, NH), 7.26 (d, 1H, *J* = 7.2Hz, ArH), 7.31-7.35 (m, 4H, ArH), 7.58 (d, 1H, CH=), 7.91(d, 1H, *J* = 6.6 Hz, ArH). EI-MS (*m/z*) 524 [M]^+^. Anal. Calcd. for C_22_H_22_ClFN_4_O_6_S: C, 50.34, H, 4.22, N, 10.67. Found: C, 50.82, H, 4.35, N, 10.32.

*(E)-methyl 2-(2-((1-(4-chloro-5-(ethylsulfonamido)-2-fluorophenyl)-3-methyl-5-oxo-1H-1,2,4-triazol-4(5H)-yl)methyl)phenyl)-3-methoxyacrylate* (**1b)**: ^1^H-NMR: δ 1.40 (t, 3H, *J* = 7.2 Hz, CH_3_), 1.92 (s, 3H, CH_3_), 3.22 (q, 2H, *J* = 7.2 Hz, CH_2_), 3.64 (s, 3H, CO_2_CH_3_), 3.88 (s, 3H, OCH_3_), 4.77 (s, 1H, CH_2_), 4.98 (s, 1H, CH_2_), 6.84 (s, 1H, NH), 7.17 (d, 1H, *J* = 7.2, ArH), 7.25-7.35 (m, 4H, ArH), 7.58 (d, 1H, CH=), 7.91 (d, 1H, *J* = 6.6Hz, ArH). EI-MS (*m/z*) 538 [M]^+^. Anal. Calcd. for C_23_H_24_ClFN_4_O_6_S: C, 51.25, H, 4.49, N, 10.40. Found: C, 51.27, H, 4.63, N, 10.06.

*(E)-methyl 2-(2-((1-(4-chloro-2-fluoro-5-(phenylsulfonamido)phenyl)-3-methyl-5-oxo-1H-1,2,4-triazol-4(5H)-yl)methyl)phenyl)-3-methoxyacrylate* (**1c**): ^1^H-NMR: δ 1.94 (s, 3H, CH_3_), 3.66 (s, 3H, CO_2_CH_3_), 3.89 (s, 3H, OCH_3_), 4.73 (s, 1H, CH_2_), 4.95 (s, 1H, CH_2_), 6.88 (s, 1H, NH), 7.12-8.01 (m, 12H, ArH, CH=). EI-MS (m/z) 587 [M]^+^. Anal. Calcd. for C_27_H_24_ClFN_4_O_6_S: C, 55.24, H, 4.12, N, 9.54. Found: C, 55.56, H, 4.36, N, 9.46.

*(E)-methyl 2-(2-((1-(2,4-dichloro-5-(methylsulfonamido)phenyl)-3-methyl-5-oxo-1H-1,2,4-triazol-4(5H)-yl)methyl)phenyl)-3-methoxyacrylate* (**1d**): ^1^H-NMR: δ 1.92 (s, 3H, CH_3_), 3.17 (s, 3H, CH_3_), 3.66 (s, 3H, CO_2_CH_3_), 3.88 (s, 3H, OCH_3_), 4.72, (s, 1H, CH_2_), 4.98 (s, 1H, CH_2_), 6.95 (s, 1H, NH), 7.58 (d, 1H, CH=), 7.17-7.40 (m, 6H, ArH), 7.85 (s, 1H, Ar); EI-MS (*m/z*) 541 [M]^+^; Anal. Calcd. for C_22_H_22_Cl_2_N_4_O_6_S: C, 48.81; H, 4.10; N, 10.35. Found: C, 49.12; H, 4.65; N, 9.94.

*(E)-methyl 2-(2-((1-(2,4-dichloro-5-(ethylsulfonamido)phenyl)-3-methyl-5-oxo-1H-1,2,4-triazol-4(5H)-yl)methyl)phenyl)-3-methoxyacrylate* (**1e**): ^1^H-NMR: δ 1.40 (t, 3H, *J* = 7.2 Hz, CH_3_), 1.91 (s, 3H, CH_3_), 3.22 (q, 2H, *J* = 7.2 Hz, CH_2_), 3.64 (s, 3H, CO_2_CH_3_), 3.88 (s, 3H, OCH_3_), 4.64, (s, 1H, CH_2_), 4.97 (s, 1H, CH_2_), 6.89 (s, 1H, NH), 7.17-7.59 (m, 6H, ArH), 7.87 (s, 1H, CH=); EI-MS (*m/z*) 555 [M]^+^; Anal. Calcd. for C_23_H_24_Cl_2_N_4_O_6_S: C, 49.74; H, 4.36; N, 10.09. Found: C, 49.95; H, 4.76; N, 9.91.

*(E)-methyl 2-(2-((1-(2,4-dichloro-5-(phenylsulfonamido)phenyl)-3-methyl-5-oxo-1H-1,2,4-triazol-4(5H)-yl)methyl)phenyl)-3-methoxyacrylate* (**1f**): ^1^H-NMR: δ 1.96 (s, 3H, CH_3_), 3.66 (s, 3H, CO_2_CH_3_), 3.88 (s, 3H, OCH_3_), 4.66, (s, 1H, CH_2_), 4.97 (s, 1H, CH_2_), 6.79 (s, 1H, NH), 7.17-8.09 (m, 12H, ArH, CH=); EI-MS (*m/z*) 603 [M]^+^; Anal. Calcd. for C_27_H_24_Cl_2_N_4_O_6_S: C, 53.74; H, 4.01; N, 9.28. Found: C, 54.06; H, 4.37; N, 9.12.

*(E)-methyl 2-(2-((1-(4-chloro-2-fluoro-5-(methylsulfonamido)phenyl)-3-methyl-5-oxo-1H-1,2,4-triazol-4(5H)-yl)methyl)phenyl)-2-(methoxyimino)acetate* (**1g**): ^1^H-NMR: δ 2.00 (s, 3H, CH_3_), 3.06 (s, 3H, CH_3_), 3.84 (s, 3H, CO_2_CH_3_), 4.07 (s, 3H, NOCH_3_), 4.77 (s, 2H, CH_2_), 6.74 (s, 1H, NH), 7.17-7.95 (m, 6H, ArH); EI-MS (*m/z*) 525 [M]^+^; Anal. Calcd. for C_21_H_21_ClFN_5_O_6_S: C, 47.96; H, 4.12; N, 13.32. Found: C, 47.63; H, 4.5; N, 13.17.

*(E)-methyl 2-(2-((1-(4-chloro-2-fluoro-5-(phenylsulfonamido)phenyl)-3-methyl-5-oxo-1H-1,2,4-triazol-4(5H)-yl)methyl)phenyl)-2-(methoxyimino)acetate* (**1h**): ^1^H-NMR: δ 2.16 (s, 3H, CH_3_), 3.87 (s, 3H, OCH_3_), 4.06 (s, 3H, NOCH_3_), 4.78 (s, 2H, CH_2_), 6.76 (s, 1H, NH), 7.17-8.03 (m, 11H, ArH); EI-MS (*m/z*) 588 [M]^+^; Anal. Calcd. for C_26_H_23_ClFN_5_O_6_S: C, 53.11; H, 3.94; N, 11.91. Found: C, 53.00; H, 4.04; N, 11.35.

*(E)-methyl 2-(2-((1-(2,4-dichloro-5-(methylsulfonamido)phenyl)-3-methyl-5-oxo-1H-1,2,4-triazol-4(5H)-yl)methyl)phenyl)-2-(methoxyimino)acetate* (**1i**): ^1^H-NMR: δ 1.99 (s, 3H, CH_3_), 3.08 (s, 3H, CH_3_), 3.84 (s, 3H, OCH_3_), 4.07 (s, 3H, NOCH_3_), 4.78 (s, 2H, CH_2_), 6.74 (s, 1H, NH), 7.17-7.95 (m, 6H, ArH); EI-MS (*m/z*) 542 [M]^+^; Anal. Calcd. for C_21_H_21_Cl_2_N_5_O_6_S: C, 46.50; H, 3.90; N, 12.91. Found: C, 46.17; H, 4.01.; N, 12.46.

*(E)-methyl 2-(2-((1-(2,4-dichloro-5-(ethylsulfonamido)phenyl)-3-methyl-5-oxo-1H-1,2,4-triazol-4(5H)-yl)methyl)phenyl)-2-(methoxyimino)acetate* (**1j**): ^1^H-NMR: δ 1.40 (t, 3H, *J* = 7.2 Hz, CH_3_), 2.01 (s, 3H, CH_3_), 3.22 (q, 2H, *J* = 7.2 Hz, CH_2_), 3.86 (s, 3H, OCH_3_), 4.08 (s, 3H, NOCH_3_), 4.77 (s, 2H, CH_2_), 6.89 (s, 1H, NH), 7.17-7.87 (m, 6H, ArH); EI-MS (*m/z*) 556 [M]^+^; Anal. Calcd. for C_22_H_23_Cl_2_N_5_O_6_S: C, 47.49; H, 4.17; N, 12.59. Found: C, 47.37; H, 4.47; N, 12.50.

*(E)-methyl 2-(2-((1-(2,4-dichloro-5-(phenylsulfonamido)phenyl)-3-methyl-5-oxo-1H-1,2,4-triazol-4(5H)-yl)methyl)phenyl)-2-(methoxyimino)acetate* (**1k**): ^1^H-NMR: δ 2.02 (s, 3H, CH_3_), 3.68 (s, 3H, OCH_3_), 3.98 (s, 3H, NOCH_3_), 4.77 (s, 2H, CH_2_), 6.75 (s, 1H, NH), 7.17-8.16 (m, 11H, ArH); EI-MS (*m/z*) 604 [M]^+^; Anal. Calcd. for C_26_H_23_Cl_2_N_5_O_6_S: C, 51.66; H, 3.84; N, 11.59. Found: C, 51.78; H, 4.05; N, 11.15.

*methyl 2-((1-(4-chloro-2-fluoro-5-(methylsulfonamido)phenyl)-3-methyl-5-oxo-1H-1,2,4-triazol-4(5H)-yl)methyl)phenyl(methoxy)carbamate* (**1l**): ^1^H-NMR: δ 2.18 (s, 3H, CH_3_), 3.105 (s, 1H, CH_3_), 3.76 (s, 3H, CO_2_CH_3_), 3.84 (s, 3H, NOCH_3_), 4.94 (s, 2H, CH_2_), 6.83 (s, 1H, NH), 7.17-7.91 (m, 6H, ArH); EI-MS (*m/z*) 514 [M]^+^; Anal. Calcd. for C_20_H_21_ClFN_5_O_6_S: C, 46.74; H, 4.12; N, 13.63. Found: C, 47.09; H, 4.41; N, 13.36.

*methyl 2-((1-(4-chloro-5-(ethylsulfonamido)-2-fluorophenyl)-3-methyl-5-oxo-1H-1,2,4-triazol-4(5H)-yl)methyl)phenyl(methoxy)carbamate* (**1m**): ^1^H-NMR: δ 1.40 (t, 3H, *J* = 7.2 Hz, CH_3_), 2.18 (s, 3H, CH_3_), 3.16 (q, 2H, *J* = 7.2 Hz, CH_2_), 3.76 (s, 3H, OCH_3_), 3.84 (s, 3H, NOCH_3_), 4.94 (s, 2H, CH_2_), 6.78 (s, 1H, NH), 7.17-7.95 (m, 6H, ArH); EI-MS (*m/z*) 528 [M]^+^; Anal. Calcd. for C_21_H_23_ClFN_5_O_6_S: C, 47.77; H, 4.39; N, 13.27. Found: C, 47.87; H, 4.63; N, 12.97.

*methyl 2-((1-(4-chloro-2-fluoro-5-(phenylsulfonamido)phenyl)-3-methyl-5-oxo-1H-1,2,4-triazol-4(5H)-yl)methyl)phenyl(methoxy)carbamate* (**1n**): ^1^H-NMR: δ 2.18 (s, 3H, CH_3_), 3.68 (s, 3H, OCH_3_), 3.86 (s, 3H, NOCH_3_), 4.95 (s, 2H, CH_2_), 6.78 (s, 1H, NH), 7.17-7.95 (m, 11H, ArH); EI-MS (*m/z*) 576 [M]^+^; Anal. Calcd. for C_25_H_23_ClFN_5_O_6_S: C, 52.13; H, 4.02; N, 12.16. Found: C, 52.32; H, 4.34; N, 12.01.

*methyl 2-((1-(2,4-dichloro-5-(methylsulfonamido)phenyl)-3-methyl-5-oxo-1H-1,2,4-triazol-4(5H)-yl)methyl)phenyl(methoxy)carbamate* (**1o**): ^1^H-NMR: δ 2.18 (s, 3H, CH_3_), 3.69 (s, 3H, OCH_3_), 3.84 (s, 3H, NOCH_3_), 4.95 (s, 2H, CH_2_), 7.05 (s, 1H, NH), 7.17-7.84 (m, 6H, ArH); EI-MS (*m/z*) 530 [M]^+^; Anal. Calcd. for C_20_H_21_Cl_2_N_5_O_6_S: C, 45.29; H, 3.99; N, 13.20. Found: C, 45.53; H, 4.25; N, 12.94.

*methyl 2-((1-(2,4-dichloro-5-(ethylsulfonamido)phenyl)-3-methyl-5-oxo-1H-1,2,4-triazol-4(5H)-yl)methyl)phenyl(methoxy)carbamate* (**1p**): ^1^H-NMR: δ 1.40 (t, 3H, *J* = 7.2 Hz, CH_3_), 2.08 (s, 3H, CH_3_), 3.19 (q, 2H, *J* = 7.2 Hz, CH_2_), 3.68 (s, 3H, OCH_3_), 3.87 (s, 3H, NOCH_3_), 6.74 (s, 1H, NH), 7.17-7.95 (m, 6H, ArH); EI-MS (*m/z*) 544 [M]^+^; Anal. Calcd. for C_21_H_23_Cl_2_N_5_O_6_S: C, 46.33; H, 4.26; N, 12.86.Found: C, 46.59; H, 4.62; N, 12.49.

*methyl 2-((1-(2,4-dichloro-5-(phenylsulfonamido)phenyl)-3-methyl-5-oxo-1H-1,2,4-triazol-4(5H)-yl)methyl)phenyl(methoxy)carbamate* (**1q**): ^1^H-NMR: δ 2.18 (s, 3H, CH_3_), 3.78 (s, 3H, OCH_3_), 3.98 (s, 3H, NOCH_3_), 4.97 (s, 2H, CH_2_), 6.84 (s, 1H, NH), 7.17-8.02 (m, 11H, ArH); EI-MS (*m/z*) 592 [M]^+^; Anal. Calcd. for C_25_H_23_Cl_2_N_5_O_6_S: C, 50.68; H, 3.91; N, 11.82. Found: C, 50.91; H, 3.97; N, 11.46.
